# Public health community engagement with Asian populations in British Columbia during COVID-19: towards a culture-centered approach

**DOI:** 10.17269/s41997-022-00699-5

**Published:** 2022-11-03

**Authors:** Wendy Pringle, Sukhmeet Singh Sachal, Gurvir Singh Dhutt, Mary Kestler, Ève Dubé, Julie A. Bettinger

**Affiliations:** 1 Vaccine Evaluation Center, BC Children’s Hospital Research Institute, Vancouver, BC Canada; 2 Faculty of Medicine, University of British Columbia, Vancouver, BC Canada; 3 Sikh Health Foundation, Surrey, BC Canada; 4 Quebec National Institute of Public Health, Québec City, QC Canada; 5 CHU de Québec-Université Laval Research Centre, Québec City, QC Canada

**Keywords:** Public health practice, Ethnic groups, Culture, Mass immunization, Infection control, Pratiques de santé publique, groupes ethniques, culture, vaccination de masse, prévention et contrôle des infections

## Abstract

**Objectives:**

COVID-19 has posed significant challenges to those who endeavour to provide equitable public health information and services. We examine how community leaders, advocates, and public health communication specialists have approached community engagement among Asian immigrant and diaspora communities in British Columbia throughout the pandemic.

**Methods:**

Qualitative interviews with 27 participants working with Asian communities in a healthcare, community service, or public health setting, inductively coded and analyzed following the culture-centred approach to health communication, which focuses on intersections of structure, culture, and agency.

**Results:**

Participants detailed outreach efforts aimed at those who might not be reached by conventional public health communication strategies. Pre-existing structural barriers such as poverty, racial disparities, and inequitable employment conditions were cited as complicating Asian diaspora communities’ experience of the pandemic. Such disparities exacerbated the challenges of language barriers, information overload, and rapidly shifting recommendations. Participants suggested building capacity within existing community service and public health outreach infrastructures, which were understood to be too lean to meet community needs, particularly in a pandemic setting.

**Conclusion:**

A greater emphasis on collaboration is key to the provision of health services and information for these demographic groups. Setting priorities according to community need, in direct collaboration with community representatives, and further integrating pre-existing bonds of trust within communities into public health communication and engagement strategies would facilitate the provision of more equitable health information and services. This mode of engagement forgoes the conventional focus on individual behaviour change, and focuses instead on fostering community connections. Such an approach harmonizes with community support work, strengthening the capacity of community members to secure health during public health emergencies.

## Résumé

**Objectifs:**

La pandémie de la COVID-19 a posé des défis importants à ceux qui visent à transmettre des informations et à offrir des services de santé publique équitables. Nous examinons comment les responsables d’organismes communautaires, les militants pour les droits et les spécialistes de la communication en santé publique ont abordé l’engagement communautaire auprès de communautés de la diaspora asiatique en Colombie-Britannique durant la pandémie.

**Méthodes:**

Des entretiens qualitatifs ont été réalisés auprès de 27 participants travaillant avec des communautés asiatiques dans un cadre de soins de santé, de services communautaires ou de santé publique. Les entretiens ont été codés et analysés de manière inductive selon l’approche centrée sur la culture de la communication en matière de santé, qui se concentre sur les intersections entre structure, culture et agencéité.

**Résultats:**

Les participants ont décrit les efforts de sensibilisation destinés à ceux qui ne sont potentiellement pas rejoints par les stratégies conventionnelles de communication en santé publique. Selon les participants, les barrières structurelles préexistantes telles que la pauvreté, les disparités raciales et les conditions d’emploi inéquitables ont aggravé l’expérience de la pandémie par les communautés de la diaspora asiatique. Ces disparités ont exacerbé les enjeux liés aux barrières linguistiques, à la surcharge d’informations et à l’évolution rapide des recommandations. Les participants ont suggéré de renforcer les capacités de communication et de sensibilisation au sein des services communautaires existants, qui étaient considérées comme trop faibles pour répondre aux besoins de la communauté, particulièrement en contexte pandémique.

**Conclusion:**

Il est essentiel d’améliorer la collaboration afin d’offrir des services et de mieux communiquer auprès de ces communautés. Pour des communications et des services de santé publique plus équitables, les besoins et les priorités doivent être identifiés en collaboration directe avec les représentants des communautés, et les relations de confiance préexistantes au sein des communautés doivent être mises à profit. Une telle approche s’harmonise avec le travail de soutien communautaire et contribuerait de façon durable à améliorer les capacités des communautés à protéger la santé pendant les urgences de santé publique.

## Introduction

Community engagement has been identified as a crucial component for public health planning (Rifkin et al., 2000; Wallerstein & Duran, 2006). It holds potential to build trust between public health leadership and communities, and to promote mutual understanding and transparency. Experts agree community engagement can have a positive impact on health outcomes in a range of settings (O’Mara-Eves et al., 2015), although there is little consensus on the best approach. For emergency planning, strong community connections can harness specific local knowledge and establish productive channels of communication (Khan et al., 2018). Engaging communities at the local level is key for establishing policies and practices that promote resilience, particularly in times of crisis.

In British Columbia, immigrant communities are predominantly East, South, and South East Asian. In the context of COVID-19, minimizing the consequences of public health interventions (such as restrictions on social gatherings, shutting down businesses and public services) on racialized communities, and addressing inequities in vaccination campaigns emerged as a key concern. In addition to growing evidence that immigrant communities in Canada have faced disproportionate hardships as a result of the COVID-19 pandemic (Statistics Canada, 2020), a spike in anti-Asian racism further stigmatized Asian residents of BC. During the first two years of the pandemic, 15% of individuals of East Asian origin reported a direct experience of a hate incident (British Columbia’s Office of the Human Rights Commissioner, 2022).

In Canada, anti-Asian public health discourse has roots that predate SARS in 2003. Both South Asian and East Asian immigrants have historically been the target of discriminatory discourse (Sellar, 2006), planning (Mawani, 2003), and public health campaigns (Leung & Guan, 2008). In the 1890s, residents of Vancouver’s Chinatown were blamed for disease outbreaks and disproportionately targeted in sanitation and vaccination campaigns (Reitmanova et al., 2015; Ward, 1990). In 1912, under the guise of public health, plans to turn away South Asian immigrants aboard the *Komagata Maru* began prior to the ship’s arrival (Wallace, 2013). Building trust through community engagement requires a good understanding of local practices as well as deep-rooted institutional and structural barriers that shape intercultural inequity today.

This study describes how community leaders and public health communication specialists approached community engagement during the COVID-19 pandemic in the Lower Mainland area of BC to identify gaps between Asian immigrant community needs and local context and public health outreach practices. Using Dutta’s culture-centered approach (CCA) (Dutta, 2008) to health communication, we explore structure, culture, and agency, three intersecting facets that illustrate how Asian communities in BC have experienced the COVID-19 pandemic and how engagement efforts aimed to provide equitable public health information and services.

## Methods

This study’s methodology is rooted in an area of scholarship known as critical health communication (CHC), which uses qualitative data to inform in-depth analysis into the ways that complex social problems are embedded within health systems. CHC treats different types of knowledges such as medical expertise, and lived experiences of illness, as “assemblages of beliefs that are created through human interaction and pre-existing meanings” (Lupton, 2003, p. 50). As with critical qualitative public health research, CHC research challenges “taken-for-granted assumptions about public health practice and research” (Mykhalovskiy et al., 2018, p. 615). Because this mode of inquiry shows how particular meanings are developed through communicative practices, it is well suited to exploring how people from different spheres of society and positionalities may interpret the same phenomena differently.

### Culture-centered approach

**Fig. 1:**
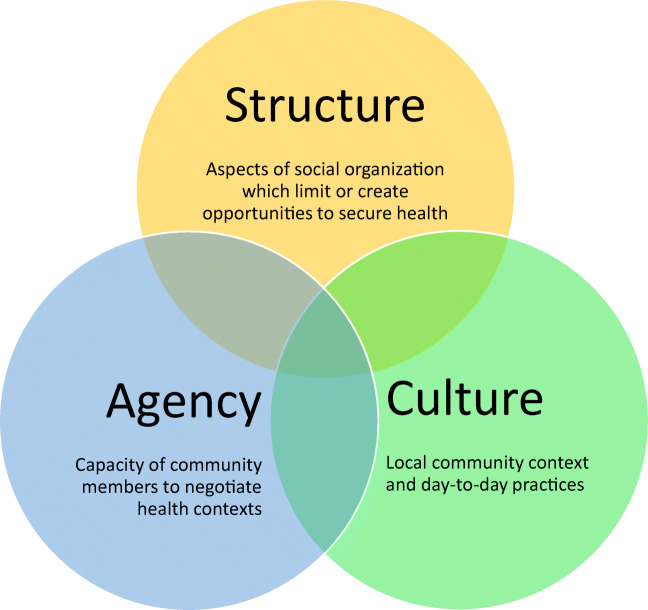
The culture-centered approach to health communication

The CCA is a guiding analytic framework that centers cultural factors. The approach examines three intersecting constructs: structure, culture, and agency (see Fig. 1). Culture is understood to be the local, meanings, definitions, and grassroots experiences of health (re)constituted through everyday interactions (Dutta, 2008; Geertz, 1973). Structures are conceptualized as patterns of social organizing, such as medical services, transportation, food, and shelter, that enable (or constrain) cultural members’ ability to access resources and engage in healthful behaviours (Dutta, 2008; Giddens, 1984; Sastry, 2016; Sastry et al., 2021; Sharma, 2009). Agency “reflects the human capacity to engage with and make sense of the cultural frameworks and structural configurations that constitute one’s social existence” (Basu, 2010; Basu & Dutta, 2008; Sastry et al., 2021, p. 381). The three constructs are understood to intersect; culture interacts with structural processes and also with agency as enacted in individual choices and actions.

The CCA’s commitment to dynamic interpretations of culture makes it appropriate for examining questions of community public health engagement. The CCA is intended as a “framework for organizing social change-oriented health interventions” (Dutta, 2008; Sastry et al., 2021, p. 386). CCA is distinct from approaches centered on tailoring health messaging or the development of culturally sensitive interventions (Kreuter et al., 2005; Noar et al., 2007) in that it does not begin from a static or a priori definition of culture, as defined by the researchers, but defines culture in research participants’ own terms. By directly engaging structural factors such as gender and institutional racism, the framework is attuned to engaging questions of social justice at play in public health policy and practice (Smith et al., 2018). While the CCA approach often engages longer in-depth narrative descriptions, in the interest of reaching more public health professionals and policy decision-makers among our readers, we have favoured a streamlined approach to sharing results. In this paper, the term culture operates in two ways: first, in reference to the CCA framework and analytic approach; and second to describe the local meanings, practices, and health experiences of study participants as they relate to agency and structural factors.

### Participants and setting

This study took place in Vancouver and its surrounding suburbs, which is one of the most diverse urban/suburban areas in Canada. East, South, and Southeast Asians are the next most populous ethnic groups after European, and Mandarin, Cantonese, and Punjabi are the most popular languages after English (Statistics Canada, 2017). Recruitment focused on Vancouver, Burnaby, Richmond, and Surrey, cities with greater concentrations of Asian diaspora communities. Key informants were purposively recruited via email, on the basis of their doing ongoing public health, clinical care, or social services work with Asian immigrant communities in the region. Recruitment was not focused on any specific professional designations, but rather on individuals with communication roles within health authority and government agencies, community support workers, or volunteers working with advocacy initiatives. The rate of response was approximately 70%.

### Data collection

Between May and November 2021, audio-recorded interviews 60 minutes in length were conducted by Zoom or telephone, according to participant preference. A semi-structured interview guide addressed participant experience with community engagement, pre-existing challenges with the health system, and community experiences of COVID-19 and public health measures. Twenty-four interviews were conducted in English and one in both Punjabi and English. Interviews were transcribed using smooth verbatim by a digital transcription service. Transcripts were checked for accuracy (audio files were replayed in full, for comparison to the transcript text) and any identifying details were removed by research staff trained in qualitative methods. Participants were then invited to review their own transcript for accuracy and privacy. Twenty participants requested to review a copy, seven declined, and one participant requested a change to their transcript. Pseudonyms were assigned to protect participant identity. All participants provided written informed consent prior to participation. This study received funding from the Canadian Institutes of Health Research (grant #: 440293) and received formal ethics approval from the UBC Children’s and Women’s Research Ethics Board.

### Data analysis

Data were analyzed according to Braun and Clarke’s method of thematic analysis to identify, analyze, and report patterns within the data (Braun & Clarke, 2006). Data analysis began with familiarization of the complete set of transcripts. Transcripts were then coded inductively with NVivo Software (QSR International) to identify passages relevant to the research topic, with two coders (GD and WP) independently generating initial codes for each of the first 12 transcripts. Similar codes and coded extracts were then reviewed and compared by both coders, collated according to key themes by GD, and the remaining 13 transcripts were coded accordingly by GD.

Theme definitions were drafted and revised by both coders to establish a best fit between data and research question (e.g., themes and definitions were reviewed and split, regrouped, or revised as appropriate). The resulting themes were subsequently mapped onto the core constructs of the CCA framework, comprising three thematic categories of structure, culture, and agency (Dutta, 2008, 2014). As such, themes were constructed both inductively in the initial stages of familiarization and coding and then deductively by way of the subsequent interpretive framing of the CCA.

## Results

Between May and November 2021, 25 interviews were conducted with 27 participants (two sets of participants requested to be interviewed as a pair). The interview period roughly corresponds with the province’s (age-based, phased approach) campaign to vaccinate the general population against COVID-19 (first and second doses). Ten participants were communication specialists employed by a health authority in an outreach or public communications capacity, 10 were practicing medical professionals with a leadership or advocacy role, seven worked in public or non-profit community service organizations, and two were volunteer community advocates. Nineteen participants were women, and eight were men. Participant age ranged from 25 to 75 years, with an average of 45. Average age did not differ by grouping. Fifteen participants spoke to their experience working with South Asian communities (predominantly Punjabi speakers and members of the Sikh community), six with East Asian communities (predominantly Chinese speakers), one with a South East Asian community (Filipino), and five with the Lower Mainland population in general.

### Uneven foundations: Structure

Participants shared examples of structural factors such as inequitable employment, transport (or lack thereof), rapidly shifting policies, and the uneven provision of existing health services that had constrained access to health prior to the pandemic, but were exacerbated by the pandemic (Table 1). Negative past experiences in healthcare settings, employment, and inequitable living conditions were cited as causing undue difficulties for community members seeking access to COVID measures such as vaccines, tests, and paid sick leave. Expanded outreach was seen as an opportunity to address confusion about rapidly shifting rules and recommendations (e.g., masking and vaccination). Community leaders emphasized that having had a negative experience such as racial discrimination or absent language interpretation in a medical setting prior to the pandemic contributed to a lack of trust in the health system in general. Public health communication specialists noted newly implemented outreach activities (such as info sessions in accessible locations) had provided the opportunity to engage groups typically difficult to reach, such as refugees.

**Table 1:** Structure

Medical services	And services aren’t always done in appropriate ways. Taking the cultural sort of safety angle of it or … in terms of language abilities. The outreach has been at many times lacking, [such as] understanding how the culture community works … [and] where they gather and going out there. (Healthcare provider and community advocate, 312)
Transport	… There wasn’t a vaccine clinic in the Downtown Eastside. The closest one was Olympic Village, and … no one who’s low income really goes to Olympic Village, they wouldn’t even know where it is, even if you give them the address. (Social services professional, 320)
Employment	…They were frontline workers working minimum wage jobs. So, I do feel that they were disproportionately affected. I know people who are working meat plants, … for a long-term care facility [as] LPNs, [or] in the trucking, taxi, or food industry. They were all adversely affected because they were in high-risk jobs, and they did not have the correct information. (Healthcare provider and community advocate, 211)
Policy/messaging	But for those who don’t quite understand why one day we said masks, and other day we said no masks. One day we said one dose, and then wait for eight weeks, wait for four weeks, wait for six months, and now wait for 28 days … And they said, “Why did I have to wait six months, then?” You can see why it’s confusing, right? (Healthcare provider and community advocate, 229)
Community outreach	…Everything is changing all the time … Like, we’re building the plane as it’s flying. We’re learning and integrating our learnings, and moving forward all the time ... [We hear from] our physicians who work with people in the community, our spokespeople, different community groups, different advisors. We are constantly getting information and scanning our environment. (Communication specialist, 074)

Communication specialists cited ever-evolving updates on best practices for engaging specific populations, difficulty finding translation services, and lengthy institutional approval processes as impeding timely delivery of outreach services and information. Community advocates and healthcare providers felt strongly that important public health outreach services (e.g., parents’ and seniors’ groups) had been cut in the years prior to the pandemic, making it virtually impossible to quickly re-establish trust between health authorities and communities during the pandemic.

### Capable connections: Culture

Cultural factors were understood to serve as both barriers and facilitators to the delivery of public health information and services (Table 2). Local channels of communication such as ethnic radio and TV stations, specific social media platforms (e.g., WeChat and WhatsApp), and trusted interpersonal connections were emphasized as key modalities to engage the community. Grassroots communication efforts focused on utilizing established bonds of trust within the cultural community to deliver public health updates and recommendations. Such strategies often focused on informal networks to both translate information into the appropriate language and localize and personalize the message.

**Table 2:** Culture

Theme	Example
Local media and communication tools	… South Asian communities—I’m talking about seniors in South Asian communities—They depend on radio. Okay? They are getting their information from radio, because they won’t go and search online. Yeah? They can’t do that. (Communication specialist, 103b)
Language differences	…A lot of the more elderly, Chinese seniors actually are illiterate and … there’s a lot of politics and nuances around the Chinese language, that has made some people illiterate … Then some of them also grew up in a time where they cut out education, or they came from villages that didn’t have schooling … And then, and then factor in eyesight and trust … Just because it’s written down doesn’t mean everyone’s going to read it. (Social services professional, 320)
Familial/religious ties	Those [who] are undocumented, refugees, or visitors … we’ve noticed that a lot of them are coming out to the “Inreach” community clinics like at the gurdwaras, mandirs, and mosques, where they feel comfortable going … And we’ve heard from the seniors making comments like, “Okay, the vaccine is being given at a place of worship … We feel more safe in receiving the vaccine.” (Communication specialist, 141)
Shared experience/trust	If you came to me and said, “[Participant], we have a vaccination site on this day at this time. You need to be there and go get it done.” I can probably respond with “Yeah, sure. [Interviewer], that sounds great. Count me in.” Right? But for other people, you have to ensure that equity, you have to really do the dance, this nuanced dance, right? Develop and nurture that bond—that trust it takes and it’s much more protracted than we would assume otherwise. (Social services volunteer, 280)

Community leaders and social services professionals noted that merely translating public health information (rather than co-creating materials) was often insufficient and viewed past consultations between community and public health as too superficial to address core communication challenges. Religious centers (such as gurdwaras, mandirs, and mosques) and familiar or culturally significant gathering spaces (like local community centers) were seen as a key mechanism for successfully engaging with community members and delivering public health information and services. Many participants stressed current public health strategies lacked knowledge of cultural practices, such as strong intergenerational ties, and participants believed such localized knowledge could be better integrated into public health strategies to promote better health outcomes for their community.

### Resilience and fragility: Agency

Participants detailed positive instances of empowerment such as networks for advocacy and mutual support, but also further disempowerment of people already in marginalizing conditions (Table 3). Several participants noted that intersections of poverty and racialization were poorly addressed in public health strategies, noting these population groups remain largely invisible, without resources or capacity to advocate for their own health. Community-led grassroots communication campaigns targeted populations with limited access to resources, such as international students, the elderly, and unsheltered populations, who were seen as more difficult to reach via mainstream communication practices and more vulnerable to misinformation.

**Table 3:** Agency

Theme	Example
Community advocacy	Well, I think for us when the numbers were up-ticking in November… and Diwali was coming and Guru Nanak’s birthday was coming. That was stressful for us, to be finger pointed and said: “Oh, its these South Asians—It’s their big fat Indian weddings.” It wasn’t a nice feeling. And then my skin color is brown... It doesn’t matter where I live or what my socio-economic status is. You’ve got to own it, and you’ve got to go: “Okay, let’s fix the situation.” (Healthcare provider and community advocate, 211)
Grassroots communication campaigns	And it was kind of ridiculous that we were acting as the call centre for … for people and collecting their information to come to this clinic. It was a success … we were able to get over 1000 people vaccinated. But it does leave a very bitter taste in my mouth, just to be honest, because of how ignored this population is. (Social services professional, 320)
Community info seeking/sharing	The rest of the seniors, they have difficulties … and some clients don’t know how to register… These are the people that we helped to register. We used to do a three-way conference. Like we would call our clients from here, and then [call] [Health Authority 2], and register them for their vaccine over the phone. (Social services professional, 245)
Health authority-community negotiation or consultation	“The health authority was really focusing on high-risk populations living in SROs [single room occupancy] and people struggling with homelessness or precarious housing. There were times where we were sending Chinese seniors who are living in SROs to vaccination clinics, and they were turned away because there was an assumption that there are no such thing as Chinese seniors living in SROs … It feels like every time there’s a new policy … We constantly have to be hounding at the door to be like, “Hey, did you forget about us?” … and it’s like screaming into the wind. (Social services professional, 320)

Countering stigmatizing narratives, such as Asian communities driving COVID transmission or resisting vaccination, was seen by community service professionals and healthcare providers as an important strategy to protect communities, as was the need for collecting disaggregated race-based data to better understand how racialized communities were disproportionately impacted by the pandemic. While communication specialists described making available lower-tech options to access health info and services (such as telephone registration for vaccine appointments), social services professionals described these measures as insufficient for those who still faced significant barriers due to poverty, isolation, and old age. Social services professionals explained that their role in supporting marginalized individuals to navigate pandemic measures was done at a major cost to other essential community support priorities, such as providing food, or navigating challenges relating to immigration, housing, poverty, and employment.

## Discussion

These findings suggest a number of important strategies that could improve the provision of equitable health information and services to culturally distinct communities and foster stronger relationships between public health and marginalized communities. Data were collected during the early months of the COVID-19 immunization campaign, a period of rapidly shifting public health policies, but suggest how community engagement practices might be improved both in ‘regular’ times and in time-constrained future public health emergency scenarios.

### Build capacity across outreach and community services

Existing community engagement strategies were stretched particularly thin in instances where structural barriers intersected with cultural factors (e.g., “Just because it’s written down doesn’t mean everyone’s going to read it”, Table 2, Language differences). For those working within a health authority in an outreach or public communications capacity, uncertainty around COVID itself proved challenging, but the added complexity of figuring out how to meet community need ‘on the fly’ and with minimal community input (e.g., “building the plane as it’s flying”) resulted in a superficial understanding of these communities, with messages and actions that did not meet community need, leading to uneven results.

As Smith et al. note, “failing to confront uncomfortable, structural injustices may therefore render work in ‘health equity’ complicit in perpetuating such inequities” (Smith et al., 2018, p. 640). Communication specialists often had a robust understanding of the challenges faced by Asian immigrant communities in the area, but the added pressures of the pandemic required constant reconfiguration of outreach work. Communication strategies had a limited capacity to remedy the structural factors constraining community members’ access to health services (e.g., lacking a health ID card or transport to a vaccine clinic). These findings suggest there is much room for growth in roles that bridge public health and social services, particularly for those with relevant cultural and linguistic fluency. With a culture-centered approach, even ordinary health promotion outreach (e.g., parents’ and seniors’ groups) can bolster confidence in public health, provide opportunities to develop localized knowledge of cultural factors, and promote mutual trust.

Participants frequently asked for community engagement with a more familiar, human face such as trusted community members or leaders. In planning for public health emergencies, community engagement should identify the strengths and assets of particular groups, rather than focusing solely on vulnerabilities (Khan et al., 2018, p. 7). Given the steep barriers to accessing healthcare in ‘regular times,’ public health strategies that account for foundational challenges (e.g., inequitable housing and employment) are better equipped to respond in times of crisis. COVID-related demands overtook the responsibilities of those working in social services and community support settings, at the expense of other supports. Building community support capacity builds trust by integrating existing trusting relationships into engagement strategies, and these relationships can then be harnessed during a crisis.

### Collaborate on equal terms

Localized knowledge of cultural factors should be at the center of strategies to engage diaspora populations. Historically, racialized communities have been at the receiving end of public health interventions and more likely to be cast as ‘resistant’ or ‘noncompliant’ (Kajeepeta et al., 2022; Reitmanova et al., 2015). These findings suggest that to promote mutual understanding and transparency between public health and cultural communities, collaboration should happen on even ground, with respect.

In order to build trust, public health priorities should be set by communities themselves. The decision to work directly with gurdwaras, mandirs, and mosques to organize vaccine clinics and disseminate COVID information exemplifies a key moment of success in these findings. In this instance, direct feedback from community advocates resulted in a rapid shift in vaccination program strategy that was widely seen to have a positive impact. In other instances, communities felt they were “screaming into the wind,” with outreach strategies being tailored too little or too late to meet community need. Community advocates and social services workers often knew what it would take to meet community needs, but felt they were either ignored or consulted too late in the process to make a difference. Open dialogue is key to engaging racialized communities (Kadambari & Vanderslott, 2021). There is no ‘one size fits all’ engagement strategy; the work to design well-suited solutions requires sufficient time and resources.

A culture-centered approach is rooted in the practice of listening, which “offers an opening for interrogating the inequities in the global landscape of power distribution, by attending to unvoiced assumptions and principles” (Dutta, 2014, p. 69). When asked what they would like public health planners to know about community engagement, several community advocates and social services workers said that humility and mutual respect were essential to productive community partnerships. Evidence shows trusting relationships are essential to the success of public health interventions like vaccination (Fahlquist, 2017; Jarrett et al., 2015). These findings suggest that there is more work to be done in building trust between communities and public health and that taking a culture-centered approach can bridge the gap.

## Conclusion

These findings suggest that a centralized or general approach to public health community engagement cannot withstand the pressures of a crisis situation such as the COVID-19 pandemic. Local, targeted, and tailored community engagement is necessary to reach diverse communities. This mode of engagement forgoes the conventional focus on individual behaviour change and focuses instead on fostering community connections. To accurately assess community need, public health authorities should work in close collaboration with community representatives. In our findings, key moments of success were driven by diverse public health teams who could harness their knowledge of local language and cultural conventions to build engagement strategies that had positive impacts. Our study explores key similarities across the experiences of a highly diverse set of Asian communities; however, future studies could more closely consider cultural factors specific to individual immigrant communities or cultural groups. Good community engagement is both time and resource intensive. To better meet the need of Canada’s diaspora and immigrant communities, both in normal situations and in times of crisis, local health authorities and the community organizations serving local populations likely need greater resources. Putting culture at the center of community engagement strategies offers key opportunities to address inequities.

## Contributions to knowledge

What does this study add to existing knowledge?

- Appropriate cultural and linguistic fluency was critical to engaging Asian diaspora communities in British Columbia during COVID, but there is room to build capacity.
- Community engagement strategies benefitted from open-dialogue partnerships that refined planning to better meet community need.
- Low-tech, in-person, and personalized communication was necessary to reach those isolated by poverty, age, and language barriers.

What are the key implications for public health interventions, practice, or policy?

- To enhance trust, recommendations should be delivered by familiar faces, such as community advocates and trusted healthcare providers who know the language and local culture.
- Public health priorities should be set in consultation with communities themselves, particularly those facing deep-rooted structural barriers.
- As health systems move to better prepare for public health emergencies, community outreach should take a culture-centered approach to promote resilience and mutual trust.
